# Supplementary material for: Heat adaptation of phage T7 under an extended genetic code
Source: Virus Evol. 2021 Dec 1;7(2):veab100. doi: 10.1093/ve/veab100 (PMC8923235; doi:10.1093/ve/veab100)
Supplement: veab100_Supp [file veab100_supp.zip › Sequencing_Analysis.zip!/Sequencing_Analysis/A1/evidence/JC_SIDE_1_57.html]

BRESEQ :: Evidence


| New junction evidence | | | | | | | | | | | |
| --- | --- | --- | --- | --- | --- | --- | --- | --- | --- | --- | --- |
|  | | seq id | position | reads (cov) | reads (cov) | score | skew | freq | annotation | gene | product |
| \* | ? | NC\_001604.1 | = 13277 | 2147 (0.700) | 671 (0.220) | 245/586 | 2.8 | 38.4% | intergenic (–/–) | *–/–* | –/– |
|  | ? | NC\_001604.1 | 14297 = | 53 (0.020) |  |  |  |  | intergenic (–/–) | *–/–* | –/– |

Only 100 of 2844 total aligned reads displayed.  

`AGGTAAAGCACATGAGGAAGGTCGCCCCGTTTCTATTACTGACCTACGTGGTTCTGGCGCACTACGCCAACTATCTGATACTATTATTGCCCTTGAGCGTAATCAGCAAGGCGATATGCCTAACCTTGTCCTCGTTCGTATTCTCAAGTGCCGCTTTACTGGTGATACTGGTATCGCTGGCTACATGGAATACAACAAGGAAACCGGATGGCTTGAACCATCAAGTTACTCAGGGGAAGAAGAGTCACACTCAGAGTCAACAGACTGGTCCAACGACACTGACTTCTGACAGGATTCTTGATGACTTTCCAGACGACTACGAGAAGTTTCGCTGGAGAGTCCCATTCTAATACGACTCACTAAAGGAGACACACCATGTTCAAACTGATTAAGAAGTTAGGCCAACTGCTGGTTCGTATGTACAACGTGGAAGCCAAGCGACTGAACGATGAGGCTCGTAAAGAGGCCACACAGTCACGCGCTCTGGCGATTCGCTCCAACGAACTGGCTGACAGTGCATCCACTAAAGTTACCGAGGCTGCCCGTGTGGCAAACCAAGCTCAACAGCTTTCCAAATTCTTTGAGTAATCAAACAGGAG  >  NC_001604.1/12977‑13575`  

                                                                                                                                                                                                                                                                                                            `|`                                                                                                                                                                                                                                                                                                            
`aGGTAAAGCACATGAGGAAGGTCGCCCCGTTTCTATTACTGACCTACGTGGTTCTGGCGCACTACGCCAACTATCTGATACTATTATTGCCCTTGAGCGTAATCAGCAAGGCGATATGCCTAACCTTGTCCTCGTTCGTATTCTCAAGTGCCGCTTTACTGGTGATACTGGTATCGCTGGCTACATGGAATACAACAAGGAAACCGGATGGCTTGAACCATCAAGTTACTCAGGGGAAGAAGAGTCACACTCAGAGTCAACAGACTGGTCCAACGACACTGACTGCTGACAGGATTCTTGA                                                                                                                                                                                                                                                                                                            >  1:137860‑M1/1‑301 (MQ=255)`  
 `ggTAAAGCACATGAGGAAGGTCGCCCCGTTTCTATTACTGACCTACGTGGTTCTGGCGCACTACGCCAACTATCTGATACTATTATTGCCCTTGAGCGTAATCAGCAAGGCGATATGCCTAACCTTGTCCTCGTTCGTATTCTCAAGTGCCGCTTTACTGGTGATACTGGTATCGCTGGCTACATGGAATACAACAAGGAAACCGGATGGCTTGAACCATCAAGTTACTCAGGGGAAGAAGAGTCACACTCAGAGTCAACAGACTGGTACAACGACACTGACTTCTGACAGGATTCTTGAc                                                                                                                                                                                                                                                                                                           <  1:258829‑M1/301‑2 (MQ=255)`  
          `aatGAGGAAGGTCGCTCCGTTTCTATTACTGACCTACGTGGTTCTGGCGCACTACGCCAACTATCTGATACTATTATTGCCCTTGAGCGTAATCAGCAAGGCGATATGCCTAACCTTGTCCTCGTTCGTATTCTCAAGTGCCGATTTACTGGTGATACTGGTATCGCTGGCTACATGGAATACAACAAGGAAACCGGATGGCTTGAACCATCAAGTTACTCAGGGGAAGAAGAGTCACACTCAGAGTCAACAGACTGGTCCAACGACACTGACTTCTGACAGGATTCTTGAcagttgtttc                                                                                                                                                                                                                                                                                                  <  1:277547‑M1/300‑11 (MQ=255)`  
                        `ccccGTTTCTATTACTGACCTACGTGGTTCTGGCGCACTACGCCAACTATCTGATACTATTATTGCCCTTGAGCGTAATCAGCAAGGCGATATGCCTAACCTTGTCCTCGTTCGTATTCTCAAGTGCCGCTTTACTGGTGATACTGGTATCGCTGGCTACATGGAATACAACAAGGAAACCGGATGGCTTGAACCATCAAGTTACTCAGGGGAAGAAGAGTCACACTCAGAGTCACCAGACTGGTCCAACGACCCTGACTTCTGACAGGATTCTTGATGACTTTCCAGACGACTACGAGaa                                                                                                                                                                                                                                                                                    >  1:226535/1‑301 (MQ=255)`  
                          `ccGTTTCTATTACTGCCCTACGTGGTTCGTGCGCACTACGCCAACTATCTTATACTATTATTGCCCTTGAGCGTAATCAGCAAGTCGATATGCCTAACCTTGTCCTCGTTCGTATTCTCAAGTGCCGCTTTACTGGTGATACTGGTATCGCTGGCTACATGAAATACAACAAGGAAACCGGATGGCTTGAACCATCAAGTTACTCAGGGGAAGAAGAGTCACACTCAGAGTCAACAGACTGGTCCAACGACACTGACTTCTGACAGGATTCTTGAcagttgtttcatatgaagagattgtt                                                                                                                                                                                                                                                                                  <  1:55714‑M1/301‑27 (MQ=255)`  
                          `ccGTTTCTATTACTGACCTGCATGGGTCTGGCGCACGACGCCAACGAGCGGATACGATTATTGCCCTTGAGCGTAATCAGCAAGGCGAGATGCCTAACCGTGTCCTCGTTCGTATGCTCAAGTGCCGCTTTACTGGTGATACGGGTAGCGCTGGCTACATGGAATACAACAAGGAAACCGGATGGCTTGAACCATCAAGTTACTCAGGGGAAGAAGAGTCACACTCAGAGTCAACAGACTGGTCCAACTACACTGACTTCTGACAGGATTCTTGATGACTTTCCAGACGACTACGAGAAGt                                                                                                                                                                                                                                                                                  <  1:230638/301‑1 (MQ=255)`  
                          `ccGTTTCTATTACTGACCTACGTGGTTCTGACGCCCTACGCCAACTATCTGATACTATTATTGCCCTTGAGCGTAATCAGCAAGGCTATATGCCTAAACTTGTCCTCTTTCGTATTCTCAAGTGCCGCTTTACTGGTGATACTTGTATCTCTGGCTACTTGGAATCCAACCAGGAAAACGGATAGCTTGAACCATCAAATTACTCAGGGAAAGAAGAGTCACACTCAGAGTCACCAGCCTGATCCAACGACAATGACTTCTGCAAACAATCTTGATGAcatgttccttctacacatttccc                                                                                                                                                                                                                                                                                  >  1:490448/1‑279 (MQ=255)`  
                              `ttCTATTACTGACCTACGTGGTTCTGGCGCACTACGCCAACTATCTGATACTATTATTGCCCTTGAGCGTAATCAGCAAGGCGATATGCCTAACCTTGTCCTCGTTCGTATTCTCAAGTGCCGCTTTACTGGTGATACTGGTATCGCTGGCTACATGGAATACAACAAGGAAACCGGATGGCTTGAACCATCAAGTTACTCAGGGGAAGAAGAGTCACACTCAGAGTCAACAGACTGGTCCAACGACACTGACTTCTGACAGGATTCTTGATGACTTTCCAgacgacctgtctcttataca                                                                                                                                                                                                                                                                              >  1:132916/1‑287 (MQ=255)`  
                              `ttCTATTACTGACCTACGTGGTTCTGGCGCACTACGCAAACTATCTGATACTATTATTGCCCTTGAGCGTAATCAGCAAGGCGATATGCCTAACCTTGTCCTCGTTCGTATTCTCAAGTGCCGCTTTACTGGTGATACTGGTATCGCTGGCTACATGGAATACAACAAGGAAACCGGATGGCTTGAACCATCAAGGTACTCAGGGGAAGAAGAGTCACACTCAGAGTCAACAGACTGGTCCAACGACACTGACTTCTGACAGGATTCTTGAcagttgtttcatatgcacagattgttaagt                                                                                                                                                                                                                                                                              >  1:52122‑M1/1‑271 (MQ=255)`  
                                    `tACTGACCTACGTGGTTCTGGCGCACTACGCCAACTATCTGATACTATTATTGCCCTTGAGCGTAATCAGCAAGGCGATATGCCTAACCTTGTCCTCGTTCGTATTCTCAAGTGCCGCTTTACTGGTGATACTGGTATCGCTGGCTACATGGAATACAACAAGGAAACCGGATGGCTTGAACCATCAAGTTACTCAGGGGAAGAAGAGTCACACTCAGAGTCAACAGACTGGTCCAACGACACTGACTTCTGACAGGATTCTTGATGACTTTCCAGACGACTACGAGAAGTTTCGCTGgag                                                                                                                                                                                                                                                                        <  1:457325/301‑1 (MQ=255)`  
                                    `tACTGACCTACGTGGTTCTGGCGCACTACGCCAACTATCTGATACTATTATTGCCCTTGAGCGTAATCAGCAAGGCGATATGCCTAACCTTGTCCTCGTTCGTATTCTCAAGTGCCGCTTTACTGGTGATACTGGTATCGCTGGCTACATGGAATACAACAAGGAAACCGGATGGCTTGAACCATCAAGTTACTCAGGGGAAGAAGAGTCACACTCAGAGTCAACAGACTGGTCCAACGACACTGACTTCTGACAGGATTCTTGATGACTTTCCAGACGACTACGAGAAGTTTCGCTGgag                                                                                                                                                                                                                                                                        <  1:178263/301‑1 (MQ=255)`  
                                    `agaggattataagagacagggCGCACTACGCCAACTATTTGATAATATTATTGCCCTTGAGCGTAATCAGCAAGGCGATATGCCTAACCTTGTCCTCGTTCGTATTCTCAAGTGCCGCTTTACTGGTGATACTGGTATCGCTGGCTACATGGAATACAACAAGGAAACCGGATGGCTTGAACCATCAAGTTACTCAGGGGAAGAAGAGTCACACTCAGAGTCAACAGACTGGTCCAACGACACTGACTTCTGACAGGATTCTTGATGACTTTCCAGACGACTACGAGAGGTTTCGCTGgag                                                                                                                                                                                                                                                                        <  1:504765/282‑1 (MQ=255)`  
                                     `aCTGACCTACGTGGTTCTGGCGCACTACGCCAACTATCTGATACTATTATTGCCCTTGAGCGTAATCAGCAAGGCTATATGCCTAACCTTGTCCTCGTTCGTATTCTCAAGTGCCGCTTTACTGGTGATACTGGTATCGCTGGCTACATGGAATACAACAAGGAAACCGGATGGCTTGAACCATCAAGTTACTCAGGGGAAGAAGAGTCACACTCAGAGTCAACAGACTGGTCCAACGCAACTGACTTCTGACAGGATTCTTGATGACTTTCCATACGACTACGAGAAGTTTCGCTGgaga                                                                                                                                                                                                                                                                       >  1:321234/1‑301 (MQ=255)`  
                                                  `gTTCTGGCGCACTACGCCAACTATCTGATACTATTATTGCCCTTGAGCGTAATCAGCAAGGCGATATGCCTAACCTTGTCCTCGTTCGTATTCTCAAGTGCCGCTTTACTGTTGATACTGGTATCGCAGGCTGCATGGAATACAACAAGGAAACCGGATGGCTTGAACCATCAAGTTACTCAGGGGAAGAAGAGTCACACTCAGAGTCAACAGACTGGTCCAACGACACTGACTTCTGACAGGATTCTTGAcagttgtttcatatgaagagattgttacgtcacgataatcaataggagaa                                                                                                                                                                                                                                                          >  1:61366‑M1/1‑251 (MQ=255)`  
                                                  `gTTCTGGCGCACTACGCCAACTATCTGATACTATTATTGCCCTTGAGCGTAATCAGCAAGGCGATATGCCTAACCTTGTCCTCGTTCGTATTCTCAAGTGCCGCTTTACTGGTGATACTGGTATCGCTGGCTACATGGAATACAACAAGGAAACCGGATGGCTTGAACCATCAAGTTACTCAGGGGAAGAAGAGTCACACTCAGAGTCAACAGACTGGTCCAACGACACTGACTTCTGACAGGATTCTTGAcagttgtttcatatgaagagattgttaagtcacgataatcaatctgtctt                                                                                                                                                                                                                                                          >  1:77109‑M1/1‑251 (MQ=255)`  
                                                       `ggCGCACTACGCCAACTATCTGATACTATTATTGCCCTTGAGCGTAATCAGCAAGGCGATATGCCTAACCTTGTCCTCGTTCGTATTCTCAAGTGCCGCTTTACTGGTGATACTGGTATCGCTGGCTACATGGAATACAACAAGGAAACTGGATGGCTTGAACCATCAAGTTACTCAGGGGAAGAAGAGTCACACTCAGAGTCAACAGACTGGTCCAACGACACTGACTTCTGACAGGATTCTTGAcagttgtttcatatgaagagattgttaagtcacgataatcaataggagaaatcaa                                                                                                                                                                                                                                                     >  1:388930‑M1/1‑246 (MQ=255)`  
                                                           `gtataagagtcagcacTGTTACTATGAGTGCCCTTGATCGTTATCAGCAAGGCGATTTGCCTACCCTTGTCCTCGTTCGTATTCTCAAGTGCCGCTTTACTGGTGATACTGGTATCGCTGGCTACATGGAATACAACAAGGAAACCGGATGGCTTGAACCATCAAGTTACTCAGGGGAAGAAGAGTCACACTCAGAGTCAACAGACTGTTCCAACGACACTGACTTCTTACAGGATTCTTGATGACTGTCCAGACGACTACGAGAAGTTTCGCTGGAGAGTCCCATTCTAATACGACGCAc                                                                                                                                                                                                                                                 <  1:273687/286‑1 (MQ=255)`  
                                                             `cTACGCCAACTATCTGATACTATTATTGCCCTTGAGCGTAATCAGCAAGGCGATATGCCTAACCTTGTCCTCGTTCGTATTCTCAAGTGCCGCTTTACTGGTGATACTGGTATCGCTGGCTACATGGAATACAACAAGTAAACCGGATGGCTTGAACCATCAAGTTACTCAGGGTAAGAAGAGTCACCCTCAGAGTCAACAGACTGGTCCAACGACACTGACTACTGACAGGATTCTTGAcagttgtttcatatgaagagattgttaagtcacgataatcaataggagaaatcaatatgat                                                                                                                                                                                                                                               >  1:183575‑M1/1‑240 (MQ=255)`  
                                                                `cGCCAACTATCTGATACTATTATTGCCCTTGAGCGTAATCAGCAAGGCGATATGCCTAACCTTGTCCTCGTTCGTATTCTCAAGTGCCGCTTTACTGGTGATACTGGTATCGCTGGCTACATGGAATACAACAAGGAAACCGGATGGCTTGAACCATCAAGTTACTCAGGGGAAGAAGAGTCACACTCAGAGTCAACAGACTGGTCCAACGACACTGACTTCTGACAGGATTCTTGATGACTTTCCAGACGACTACGAGAAGTTTCGCTGGAGAGTCCCATTCTAATACGACTCACTAAAg                                                                                                                                                                                                                                            >  1:14573/1‑301 (MQ=255)`  
                                                                 `ttgtataagagacagcTATTATTGCCCTTGAGCGTAATCAGCAAGGCGATATGCCTAACCTTGTCCTCGTTCGTATTCTCAAGTGCCGCTTTACTGGTGATACTGGTATCGCTGGCTACATGGAATACAACAAGGAAACCGGATGGCTTGAACCATCAAGTTACTCAGGGGAAGAAGAGTCACACTCAGAGTCAACAGACTGGTCCAACGACACTGACTTCTGACAGGATTCTTGATGACTTTCCATACGACTACGAGAAGTTTCGCTGGAGAGTCCCATTCTAATACGACTCACTAAAgg                                                                                                                                                                                                                                           <  1:121819/286‑1 (MQ=255)`  
                                                                 `gagacagaTCTGATACTATTATTGCCCTTGAGCGTAATCAGCAAGGCGATATGCCTAACCTTGTCCTCGTTCGTATTCTCAAGTGCCGCTTTACTGGTGATAATGGTATCGCTGGCTACATGGAATACAACAAGGAAACCGGATGGCTTGAACCATCAAGTTACTCAGGGGAAGAAGAGTCACACTCAGAGTCAACAGACTGGTCCAACGACACTGACTTCTGACAGGATTCTTGATGACTTTCCAGACGACTACGAGAAGTTTCGCTGGAGAGTCCCATTCTAATACGACTCACTAAAgg                                                                                                                                                                                                                                           <  1:444967/294‑1 (MQ=255)`  
                                                                 `atgtgtataagagacagaatattGCCCGTGAGCGTAATCAGCAAGGCGATATGCCTAACCTTGTCCTCGTTCGTATTCTCAAGTGCCACTTTACTGGTGATACTGGTATCGCTGGCTACATGGAATACAACAAGGAAACCGGATGGCTTGAACCATCAAGTTACTCAGGGGAAGAAGAGTCACACTCAGAGTCAACAGACTGGTCCAACGACACTGACTTCTGACAGGATTCTTGATGACTTTCCAGACGACTACGAGAAGTTTCGCTGGAGAGTCCCATTCTAATACGACTCACTAAAgg                                                                                                                                                                                                                                           <  1:125572/282‑1 (MQ=255)`  
                                                                 `aagacagtacTGATACTATTATTTCCCTTGAACGTAATCAGTAAGGCGATATGCCTAACCTTGTCCTCGTTCGTATTCTCAAGTGCCGCTTTACTGGTGATACTGGTATCGCTGGCTACATGGAATACAACAATGAAACCGGATTGCTTGAACCATCAAGTTACTCAGGAGAAGAAGAGTCACACTCAGAGTCAACAGACTGGTCCAACGACACTGACTTCTGACAGGATTCTTGATGACTTTCCAAACGACTACGAGAATTTTCGCTGGAGAGTCCCATTCTAATACGACTCACTAAAgg                                                                                                                                                                                                                                           <  1:23789/292‑1 (MQ=255)`  
                                                                 `aaagggtataagagacagttattGCCCTTGAGCGTAATCAGCAAGGCGATATGCCTAACCTTGTCCTCGTGCGTATTCTCAAGTGCCGAAGTACTGGTGATACTGGTATCGCTGGCTACATGGAATACAACAAGGAAACCGGATGGCTTGAACCATCAAGTTACTCAGGGGAAGAAGAGTCACACTCAGAGTCAACAGACTGGTCCAACGACAATGACTTCTGACAGGATTCTTGATGACTTTCCAGACGACTACGAGAAGTTTCGCTGGAGAGTCCCATTCTAATACGACTCACTAAAgg                                                                                                                                                                                                                                           <  1:470503/283‑1 (MQ=255)`  
                                                                  `ccAACTATCTGATACTCTTATTGCCCTTGAGCGTAATCAGCAAGGCCATATGCCTAACCTTGTCCTCGTTCGTATTCTAAAGTGCCGCTTTACTGGTGATACTGGTATCGCTGGCTACATGGAATACAACAAGGAAACCGGATGGCTTGAACCATAAAGTAACTCAGGGGAAGAAGAGTCACACCCAGAGTCAACAGACTGGTCCAACGACACTGACTTCTGACAGGATTCTTGATGACTTTCCAGACGACTACGAGAAGTTTCGCTGGAGAGTCCCATTCTAATCCGACTCACTACAggg                                                                                                                                                                                                                                          >  1:30083/1‑300 (MQ=255)`  
                                                                   `aaacttacTGATACTATTATTGCCCTTGCGCGTAATTCGCAAGTCGATAGGCCTAACCGTCTCCTCGTCCGTATTACCAAGTGCCGCTTTACTGTTGTTACTGGTTTCGCTGGCTACATGGAATACAACAAGGAAAACGGATGGCTTGAACCATCAAGTTACTCAGGGGAAGAAGAGTCACACTCAGAGTCAACAGACTGGTCCAACGACACTGACTTCTGACAGGATTCTTGATGACTTTCCAGACGACTACGAGAAGTTTCGCTGGAGAGTCCCATTCTAATACGACTCACTAAAGgag                                                                                                                                                                                                                                         <  1:159183/294‑1 (MQ=255)`  
                                                                     `aatgtCTGGTACTATTAGTGCCCTGTAGCGAAATCAGCAAGGTGATATGCCTAACCTTGTCCTCGTTCGTATTCTCAAGTGCAGCTTTACTGGTGATACTGGTATCGCTGGATACATGGAATACAACAAGGAAACCGGATGGCTTGAACCATCAAGTTACTCAGGGGAAGAAGAGTCACACTCAGAGTCAACAGACTGGTCCAACGACACTGACTTCTGACAGGATTCTTGATGACTTTCCAGACGACTACGAGAAGTTTCGCTGGAGAGTCCCATTCTAATACGACTCACTAAAGGAGac                                                                                                                                                                                                                                       <  1:371425/297‑1 (MQ=255)`  
                                                                        `taagagacagattattgcccttgagcGTAATCAGCAAGGCGATATGCCTAACCTTGTCCTCGTTCGTATTCTCAAGTGCCGCTTTACTGGTGATACTGGTATCGCTGGCTACATGGAATACAACAAGGAAACCGGATGGCTTGAACCATCAAGTTACTCAGGGGAAGAAGAGTCACACTCAGAGTCAACAGACTGGTCCAACGACACTGACTTCTGACAGGATTCTTGAcagttgtttcatatgaagagattgttaagtcacgataatcaaacaggagaaatcaatatgatcgtttctgac                                                                                                                                                                                                                                    <  1:477915‑M1/291‑73 (MQ=255)`  
                                                                        `aTCTGATACTATTATTGCCCTTGAGCGTAATCAGCAAGGCGATATGCCTAACCTTGTCCTCGTTCGTATTCTCAAGTGCCGCTTTACTGGTGATACTGGTATCGCTGGCTACATGGAATACAACAAGGAAACCGGATGGCTTGAACCATCAAGTTACTCAGGGGAAGAAGAGTCACACTCAGAGTCAACAGACTGGTCCAACGACACTGACTTCTGACAGGATTCTTGATGACTTTCCAGACGACTACGAGAAGTTTCGCTGGAGAGTCCCATTCTAATACGACTCACTAAAGGAGacccc                                                                                                                                                                                                                                    >  1:205826/1‑298 (MQ=255)`  
                                                                                `cTATTATTGCCCTTTAGCGTAATCAGCAAGGCGATATGCCTAACCTTGTCCTCGTTCGTATTCTCAAGTGCCGCTTTACTGGTGATACTGGTATCTCTGGCTACATGGAATACAACAAGGAAACCGGATGGCTTGAACCATCAAGTTCCTCAGGGGAAGAAGAGTCACACTCAGAGTCAACAGACTGGTCCAACGACACTGCCTTCTGACAGGATTCTTGATGACTTTCCAGACGACTACGAGAAGTTCCGCTGGAGAGTCCCATTCTAATACGACTCACTAAAGGAGACACACCTTGttg                                                                                                                                                                                                                            >  1:418410/1‑300 (MQ=255)`  
                                                                                `cTATTATTGCCCTTGAGCGTAATCAGCAAGGCGATGTGCCTAACCTTGTCCTCGTTCGTATTCTCAAGTGCCGCTTTACTGGTGATACTGGTATCGCTGGCTACATGGAATACAACAAGGAAACCGGATGGCTTGAACCATCAAGTTACTCAGGGGAAGAAGAGTCACACTCAGAGTCAACAGACTGGTCCAACGACACTGACTTCTGACAGGATTCTTGATGACTTTCCAGACGACTACGAGAAGTTTCGCTGGAGAGTCCCATTCTAATACGACTCACTAAAGGAGACCCACCATGTTc                                                                                                                                                                                                                            >  1:117037/1‑301 (MQ=255)`  
                                                                                `cTATTATTGCCCTTGAGCGTAATCAGCAAGGCGATATGCCTAACCTTGTCCTCGTTCGTATTCTCAAGTGCCGCTTTACTGGTGATACTGGTATCGCTGGCTACATGGAATACAACAAGGAAACCGGATGGCTTGAACCATCAAGTTACTCAGGGGAAGAAGAGTCACACTCAGAGTCAACAGACTGGTCCAACGACACTGACTTCTGACAGGATTCTTGATGACTTTCCAGACGACTACGAGAAGTTTCGCTGGAGAGTCCCATTCTAATACGACTCACTAAAGGAGACACACCATGttt                                                                                                                                                                                                                            >  1:4312/1‑300 (MQ=255)`  
                                                                                `cTATTATTGCCCTTGAGCGTAATCAGCAAGGCGATATGCCTAACCTTGTCCTCGTTCGTATTCTCAAGTGCCGCTTTACTGGTGATACTGGTATCGCTGGCTACATGGAATACAACAAGGAAACCGGATGGCTTGAACCATCAAGTTAATCAGGGGAAGAAGAGTCACACTCAGAGTCAACAGACTGGTACTACGACACTGACTTCTGACAGGATTCTTGATGACTTTCCAGACGACTACGAGAAGTTTCGCTGGAGAGTCCCATTCTAATACGACTCACTAAAGGAGAAACACCATGTTc                                                                                                                                                                                                                            >  1:106789/1‑301 (MQ=255)`  
                                                                                  `attattGCCCTTGAGCGTAATCAGCAAGGCGATATGCCTAACCTTGTCCTCGTTCGTATTCTCAAGTGCCGCTTTACTGGTGATACTGGTATCGCTGGCTACATGGAATACAACAAGGAAACCGGATGGCTTGAACCCTCAAGTTACTCAGGGGAAGAAGAGTCACACTCAGAGTCAACAGACTGGTCCAACGACACTGACTTCTGACAGGATTCTTGATGACTTTCCAGCCGACTACGAGAAGTTTCGCTGGAGAGTCCCATTCTATTACGACTCACTAAAGGAGACACAcctgtctctt                                                                                                                                                                                                                          >  1:234340/1‑293 (MQ=255)`  
                                                                                    `tattGCCCTTGAGCGTAATCAGCAAGGCGATATGCCTAACCTTGTCCTCGTTCGTATTCTCAAGTGCCGCTTTACTGGTGATACTGGTATCGCTGGCTACATGGAATACAACAAGGAAACCGGATGGCTTGAACCATCAAGTTACTCAGGGGAAGAAGAGTCACACTCAGAGTCAACAGACTGGTCCAACGACACTGACTTCTGACAGGATTCTTGATGACTTTCCAGACGACTACGAGAAGTTTCGCTGGAGAGTCCCATTCTAATACGACTCACTAAAGGAGACACACCATGTTCAAAc                                                                                                                                                                                                                        <  1:214377/301‑1 (MQ=255)`  
                                                                                    `tagtgaccTTGAGATGAAACATCAATGAGATGTGTCTAACCTTTTCCTCGTTCGTATTCGAAAGTGCCGCTTTACTGGTGATAATAGTATCGCTGGATAAATGGAAAACAACAAGGGAACCGGAGGGCTTTAACCATCAAGTTATTCAGGGGAATAAGAATGACACTCAGAGTCAACAGACTGGTCCAACGACACTGACTTCTGACAGGATTCTTGATGACTTTCCAGACGACTACGAGAAGTTTCGCTGGAGAGTCCCATTCTACTACTACTCACGAAAGGAGACACACCATGTTCAAAc                                                                                                                                                                                                                        <  1:449142/295‑1 (MQ=255)`  
                                                                                      `ttGCCCTTGAGCGTAATCAGCAAGGCGATATGCCTAACCTTGTCCTCGTTCGTATTCTCAAGTGCCGCTTTACTGGTGATACTGGTATCGCTGGCTACATGGAATACAACAAGGAAACCGGATGGCTTGAACCATCAAGTTACTCAGGGGAAGAAGAGTAACACTCAGAGTCAACAGACTGGTCCAACGACACTGACTTCTGACAGGATTCTTGAcagttgtttcatatgaagagattgttaagtcacgataatcaataggagaaatcaatatgatcgtttctgacatcgaagctaacgcc                                                                                                                                                                                                                      >  1:8194‑M1/1‑215 (MQ=255)`  
                                                                                         `cccTTGAGCGTAATCAGCAAGGCGATATGCCTAACCTTGTCCTCGTTCGTATTCTCAAGTGCCGCTTTACTGGTGATACTGGTATCGCTGGCTACATGGAATACAACAAGGAAACCGGATGGCTTGAACCATCAAGTTACTCAGGGGAAGAAGAATCACACTCAGATTCAACAGACTGGTCCAACGACACTGACTTCTGACAGGATTCTTGATGACTTTCCAGACGACTAAGAGAAGTTTCGCTGGAGAGTCACATTCTAATACGACTTAATAAAGGAGTCACACCATGTTCAAAATGAtt                                                                                                                                                                                                                   >  1:364259/1‑301 (MQ=255)`  
                                                                                               `agcttcatcagcaAGGCGATATGCCTAACCGTGTCCTCGTTCGTATTCTCAAGTGCCGCTTTACTGGTGATACTGGTATCGCTGGCTACATGGAATACAACAAGGAAACCGGATGGCTTGAACCATCAAGTTACTCAGGGGAAGAAGAGTCACACTCCGAGTCAACAGACTGGTCCAACGACACTGACTTCTGACAGGATTCTTGAcagttgtttcatatgaagagattgttaagtcacgataatcaataggagaaatcaatatgatcgtttctgacatcgaagctaacgccctcttagag                                                                                                                                                                                                             <  1:464766‑M1/295‑96 (MQ=255)`  
                                                                                               `accgtacacatcAAGGCGATATGCCTAACCTTGTCCTCGTTCGTATTATCAAGTGCCGCTTTACTGGTGATACTGGTATCGCTGTCTACATGGAATACAACAAGGAAACAGGATGGCTTGAACCATCAAGTTACTCAGGGGAAGAAGAGTCACACTCAGAGTCAACAGACTGGTCCAACGACACTGACTTCTGACAGGATTCTTGATGACTTTCCAGCCGACTACGAGAAGTTTCGCTGTAGAGTCCCATTCTAATACGACTCACTAAAGGAGACACACCATGTTCAAACTGATTaagaag                                                                                                                                                                                                             <  1:242570/290‑1 (MQ=255)`  
                                                                                               `aGCGTAATCAGCAAGGCGATATGCCTAACCTTGTCCTCGTTCGTATTCTCAAGTGCCGCTTTACTGGTGATACTGGTATCGCTGGCTACATGGAATACAACAAGGAAACCGGATGGCTTGAACCATCAAGTTACTCAGGGGAAGAAGAGTCACACTCAGAGTCAACAGACTGGTCCAACGACACTGACTTCTGACAGGATTCTTGAcagttgtttcatatgaagagattgttaagtcacgataatcaataggagaaatcaatatgatcgtttctgacatcgaagctaacgccctcttagag                                                                                                                                                                                                             <  1:439800‑M1/301‑96 (MQ=255)`  
                                                                                                 `ggtAAGCAGCAGGGCGATATGCCTAACCTTTTACACTTTCTTATGCTCAAGTGACGCTTGACTGGTGATACTGGTATCGCTGGCTACATGGAATACAACAAGGAAACAGAATGGCTTGAACCATCAAGTTACTCAGGGGAAGAAGAGGCACACTCAGAGGCAACAGACTGGTCCAACGTCACTGACTTCTGACAGGATTCTTGAcagttgtttcatatgaagagattgttaagtcacgatactcaatagaagaaatcaatatgatcgtttctgacatcgaagctaacgccctcttagagag                                                                                                                                                                                                           <  1:95721‑M1/300‑98 (MQ=255)`  
                                                                                                 `agtAATCAGCAAGGCGATATGCCTAACCTTGTCCTCGTTCGTATTCTCAAGTGCCGCTTTACTGGTGATACTGGTATCGCTGGCTACATGGAATACAACAAGGAAACCGGATGGCTTGAACCATCAAGTTACTCAGGGGAAGAAGAGTCACACTCAGAGTCAACAGACTGGTCCAACGACACTGACTTCTGACAGGATTCTTGAcagttgtttcatatgaagagattgttaagtcacgataatcaataggagaaatcaatatgatcgtttctgacatcgaagctaacgccctcttagagag                                                                                                                                                                                                           <  1:269018‑M1/300‑98 (MQ=255)`  
                                                                                                  `gTAATCAGCAAGGCGATATGCCTAACCTTGTCCTCGTTCGTATTCTCAAGTGCCGCTTTACTGGTGATACTGGTATCGCTGGCTACATGGAATACAACAAGGAAACCGGATGGCTTGAACCATCAAGTTACTCAGGGGAAGAAGAGTCACACTCAGAGTCAACAGACTGGTCCAACGACACTGACTTCTGACAGGATTCTTGATGACTTTCCAGACGACTACGAGAAGTTTAGCTGGAGAGTCCCATTCTAATACGACTCACTAAAGGAGACACACCATGTTCAAACTGATTAAGAAGTTa                                                                                                                                                                                                          >  1:359015/1‑301 (MQ=255)`  
                                                                                                    `aaTCAGGAAGGATAGATGCCTAACCTTGTCCTCGTTCGTATGTTTAAGAGCCGCGTTACTGGTGAGACTGGTATCGCTGGCTACATGGAATACAACAAGGAAACCTGATGGCTTGAACCATCAAGTTACTCAGGGGAAGAAGAGTCACACTCAGAGTCAACAGACTGGTCCAACGACACTGACTTCTGACAGGATTCTTGATTACTTTCCAGACGACTACGAGAAGTTTCGCTGGAGAGTCCCATTCTAATACGACTCACTAAAGGAGACACACCATGTTCAAACTGATTAAGAAGTTAgg                                                                                                                                                                                                        <  1:497550/301‑1 (MQ=255)`  
                                                                                                            `aggctgtatGCCTAACCTTGGCCTCGTTCGTATTCTCAAGGTGCGCTTTACTGGTGAAACTGGTATCGCTTGCTACATGGAATACAACAAGGAAACCGGATGGCTTGAACCATCAAGTTACTCAGGGGAAGAAGAGTCACACTCAGAGTCAACAGACTGGTCCAACGACACTGACTTCTGACAGGATTCTTGATGACTTTCCAGACGACTACGAGAAGTTTCGCTGGAGAGTCCCATTCTAATACGACTCACTAAAGGAGACACACCATGTTCAAACTGATTAAGAAGTTAGGCCAActgc                                                                                                                                                                                                <  1:348443/295‑1 (MQ=255)`  
                                                                                                              `gCGATATGCCTAACCTTGTCCTCGTTCGTATTCTCAAGTGCCGCTTTACTGGTGATACTGGTATCGCTGGCTACATGGAATACAACAAGGAAACCGGATGGCTTGAACCATCAAGTTACTCAGGGGAAGAAGAGTCACACTCAGAGTCAACAGCCTGGTCCAACGACACTGACTTCTGACAGGATTCTTGATGACTTTCCAGACGACTACGAGAAGTTTCGCTGGAGAGTCCCATTCTAATACTACTCACTAAAGGAGACACACCATGTTCAAACTGATTACGAAGTTAGGCAAActgctg                                                                                                                                                                                              >  1:196511/1‑301 (MQ=255)`  
                                                                                                                 `atatGCCTAACCTTGTCCTCGTTCGTATTCTCAAGTGCCGCTTTACTGGTGATACTGGTATCGCTGGCTACATGGAATACAACAAGGAAACCGGATGGCTTGAACCATCAAGTTACTCAGGGGAAGAAGAGTCACACTCAGAGTCAACAGACTGGTCCAACGACACTGACTTCTGACAGGATTCTTGATGACTTTCCAGACGACTACGAGAAGTTTCGCTGGAGAGTCCCATTCTAATACGACTCACTAAAGGAGACACACCATGTTCAAACTGATTAAGAAGTTAGGCCAACTGCTGGtt                                                                                                                                                                                           <  1:69197/301‑1 (MQ=255)`  
                                                                                                                   `ctcggagatgtgtataagagacagattctcaagtgccgctttactggtgATACTGGTATCGCTGGCTACATGGAATACAACAAGGAAACCGGATGGCTTGAACCATCAAGTTACTCAGGGGAAGAAGAGTCACACTCAGAGTCAACAGACTGGTCCAACGACACTGACTTCTGACAGGATTCTTGAcagttgtttcatatgaagagattgttaagtcacgataatcaataggagaaatcaatatgatcgtttctgacatcgaagctaacgccctcttagagagcgtcactaagttacactg                                                                                                                                                                                         <  1:92220‑M1/277‑116 (MQ=255)`  
                                                                                                                    `tGCCTAACCTTGTCCTCGTTCGTATTCTCAAGTGCCGCTTTACTGGTGATACTGGTATCGCTGGCTACATGGAATACAACAAGGAAACCGGATGGCTTGAACCATCAAGTTACTCAGGGGAAGAAGAGTCACACTCAGAGTCAACAGACTGGTCCAACGACACTGACTTCTGACAGGATTCTTGAcagttgtttcatatgaagagattgttaagtcacgataatcaataggagaaatcaatatgatcgtttctgacatcgaagctaacgccctcttagagagctgtctcttatacacatct                                                                                                                                                                                        >  1:423459‑M1/1‑185 (MQ=255)`  
                                                                                                                     `gCCTAACCTTGTCCTCGTTCGTATTCTCAAGTGCCGCTTTACTGGTGATACTGGTATCGCTGGCTACATGGAATACAACAAGGAAACCGGATGGCTTGAACCATCAAGTTACTCAGGGGAAGACGAGTCACACTCAGAGTCAACAGACTGGTCCAACGACACTGACTTCTGACAGGATTCTTGATGACTTTCCAGACGACTACGAGAAGTTTCGCTGGAGAGTCCCATTCTAATACGACTCACTAAAGTAGACACACCATGTTCACACTGATTAAGAAGTTAGGCCAACTGCTGGTTCGtt                                                                                                                                                                                       >  1:65103/1‑300 (MQ=255)`  
                                                                                                                     `gCCTAACCTTGTCCTCGTTCGTATTCTCAAGTGCCGCTTTACTGGTGATACTGGTATCGCTGGCTACATGGAATACAACAAGGAAACCGGATGGCTTGAACCATCAAGTTACTCAGGGGAAGAAGAGTCACACTCAGAGTCAACAGACTGGTCCAACGACACTGACTTCTGACAGGATTCTTGAcagttgtttcatatgaagagattgttaagtcacgataatcaataggagaaatcaatatgatcgtttctgacatcgaagctaacgccctcttagagagcgtcactaagttccactgcg                                                                                                                                                                                       >  1:408474‑M1/1‑184 (MQ=255)`  
                                                                                                                     `gCCTAACCTTGTCCTCGTTCGTATTCTCAAGTGCCGCTTTACTGGTGATACTGGTATCGCTGGCTACATGGAATACAACAAGGAAACCGGATGGCTTGAACCATCAAGTTACTCAGGGGAAGAAGAGTCACACTCAGAGTCAACAGACTGGTCCAACGACACTGACTTCTGACAGGATTCTTGATGACTTTCCAGACGACTACGAGAAGTTTCGCTGGAGAGTCCCATTCTAATACGACTCACTAAAGGAGACACACCATGTTCAAACTGATTAAGAAGTTAGGCCAACTGCTGGTTCGTa                                                                                                                                                                                       <  1:8908/301‑1 (MQ=255)`  
                                                                                                                      `gcTAAACTTGTCATCGTTCGTAATATCAAGTGCAGCTGTAAAGGGGATACTGGGATCGCTGGCTACATGGAAGACAACAAGGAAATCGGATGGCTTGAAACATCAAGTTACGAAGGGGAAGAAGAGTCACAATAAGAGTCAACAGACTGGTCCAACGACAATGACTTCTGACAGGATTCTTGAAGACTTTCCAGACGACTACGAGAAGTTTCGCTGGAGAGGAACAGTATAATACGACTCACTAAAGGAGAAACACCATGTTCAAACTGATTAAGAAGTTAGGCCAACTGCTGGTTCGTAt                                                                                                                                                                                      <  1:253741/300‑1 (MQ=255)`  
                                                                                                                          `aCCTTGTCCTCGTTCGTATGCTCAAGTGCCGCTTTACTGGTGATACTGGTATCGCTGGCTACATGGAATACAACAAGGAAAACGGATGGCTTGAACCATCAAGTTACTCATGGGAAGAAGAGTCACACGCAGAGTCAACAGACTGGTCCAACGACACTGACTTCTGACAGTATTCTTGATGACTTTCCAGACGACTACGAGAAGTTTCGCTGGAGAGTCCCATTCTAATACGACTCACTAAAGGAGACACACCATGTTCAAACTGATTAAGAAGTTAGGCCAACTGCTGGTTCGTATGTAc                                                                                                                                                                                  <  1:187106/301‑1 (MQ=255)`  
                                                                                                                                   `tcgttcgtATTCTCAAGTGCCGCTTTACTGGTGATACTGGTATCGCTGGCTACATGGAATACAACAAGGAAACCGGATGGCTTGAACCATCAAGTTACTCAGGGGAAGAAGAGTCACACTCAGAGTCAACAGACTGGTCCAACGACACTGACTTCTGACAGGATTCTTGAcagttgtttcatatgaagagattgttaagtcacgataatcaataggagaaatcaatatgatcgtttctgacatcgaagctaacgccctcttagagagcgtcactaagttccactgcggggttatctacgac                                                                                                                                                                         <  1:412821‑M1/301‑132 (MQ=255)`  
                                                                                                                                              `ctcAAGTGCCGCTTTACTGGTGATACTGGTATCGCTGGCTACATGGAATACAACAAGGAAACCGGATGGCTTGAACCATCAAGTTACTCAGGGGAAGAAGAGTCACACTCAGAGTCAACAGACTGGTCCAACGACACTGACTTCTGACAGGATTCTTGAcagttgtttcatatgaagagattgttaagtcacgataatcaataggagaaaccattatgatcgtttctgacatcgaagctaacgccctcttagagagcgtcactaagttccactgcggggttatctacgactactccaccgc                                                                                                                                                              >  1:19076‑M1/1‑159 (MQ=255)`  
                                                                                                                                                       `cGCTTTACTGGGGATACTGGTATCGATGGCTACATGGAATACAACAAGGAAACCGGATGGCTTGAACCATCAAGTTACTCAGGGGAAGAAGAGTCACACTCAGAGTCAACAGACTGGTCCAACGACACTGACTTCTGACAGGATTCTTGATGACTTTCCAGACGACTACGAGAAGTTTCGCTGGAGAGTCCCATTCTAATACGACTCACTAAAGGAGACACACCATGTTCAAACTGATTAAGAAGTTAGGCCAACTGCTGGTTCGTATGTACAACGTGGAAGCCAAGCGACTGAACGATGa                                                                                                                                                     <  1:474478/301‑1 (MQ=255)`  
                                                                                                                                                       `agcgatACCGGTGATACTGGTATCGCTGGCTACATGGAATACAACAAGGAAACCGGATGGCTTGAACCATCAAGTTACTCAGGGGAAGAAGAGTCACACTCAGAGTCAACAGACTGGTCCAACGACACTGACTTCTGACAGGATTCTTGATGACTTTCCAGACGACTACGAGAAGTTTCGCTGGAGAGTCCCATTCTAATACGACTCACTAAAGGAGACACACCATGTTCAAACTGATTAAGAAGTTAGGCCAACTGCTGGTTCGTATGTACAACGTGGAAGCCAAGCGACTGAACGATGa                                                                                                                                                     <  1:10070/296‑1 (MQ=255)`  
                                                                                                                                                         `cTTTACTGGTGATACTGGTATCTCTGGCAACATGGAATACAACAAGGAAACCGGATGGCTTGAACCATCAAGTTACTCAGGGGAAGAAGAGTCACACTCAGAGTCAACAGACTGGTCCAACGACACTGACTTCTGACAGGATTCTTGATGACTTTCCAGACGACTACGAGAAGTTTCGCTGGAGAGTCCCATTCTAATACGACTCCCTAAAGGAGACACACCATGTTCAAACTGATTAAGAAATTAGGCCAACTGCTGGTTCGTATGTACAACGTGGAAGCCAAGCGACTGAACGATGAgg                                                                                                                                                   >  1:73565/1‑301 (MQ=255)`  
                                                                                                                                                           `ttACTGGTGATACTGGTATCGCTGGCTACATGGAATACAACAAGGAAACCGGATGGCTTGAACCATCAAGTTACTCAGGGGAAGAAGAGTCACACTCAGAGTCAACAGACTGGTCCAACGACACTGACTTCTGACAGGATTCTTGATGACTTTCCAGACGACTACGAGAAGTTTCGCTGGAGAGTCCCATTCTAATACGAATCACTAAAGGAGACACACCATGTTTAAACTGATTAAGAAGTTAGGACAACTGCTGGTTAGTATGTACAAAGTGGAAGCCACGCGACTGAAGGATGactgt                                                                                                                                                 >  1:332318/1‑297 (MQ=255)`  
                                                                                                                                                             `aCTGGTGATACTGGTATCGCTGGCTACATGGAATACAACAAGGAAACCGGATGGCTTGAACCATCAAGTTACTCAGGGGAAGAAGAGTCACACTCAGAGTCAACAGACTGGTCCAACGACACTGACTTCTGACAGGATTCTTGATGACTTTCCAGACGACTACGAGAAGTTTAGCTGGAGAGTCCCATTCTAATACGACTCACTAAAGGAGACACACCATGTTCAAACTGATTAAGAAGTTAGGCCAACTGCTGGTTAGTATGTACAACGTAGCAGCAAAGCGACTGAACGATGAGGCTcc                                                                                                                                               >  1:48234/1‑300 (MQ=255)`  
                                                                                                                                                                `tggctcggagatgtgtgtaagagacaggAATACAACAAGGAAACTGGTTGGCTTGACCCATCAAGTTTCTCAGGGGAAGAAGAGTCACACTCATAGTCAACAGACTTGTCCAACGACACGGACTTCTGACAGGATTCTTGATGACTTTCCAGTCGACTACGAGAAGTTTCGCTGGAGAGTCCCATTCTAATTCGACTCACTAAAGGAGACACACCCTGTTCAAACTGATTAAGAAGTTAGGCCAACTGCTGGTTCGTATGTACAACGTGGAAGTCAAGCGACTGAACGATGAGGCTCGTaa                                                                                                                                            <  1:34270/275‑1 (MQ=255)`  
                                                                                                                                                                `ggTGATACTGGTATCGCTGGCTACATGGAATACAACAAGGAAACCGGATGGCTTGAACCATCAAGTTACTCAGGGGAAGAAGAGTCACACTCAGAGTCAACAGACTGGTCCAACGACACTGACTTCTGACAGGATTCTTGATGACTTTCCAGACGACTACGAGAAGTTTCGCTGGAGAGTCCCATTCTAATACGACTCACTAAAGGAGACACACCATGTTCAAACTGATTAAGAAGTTAGGCCAACTGCTGGTTCGTATGTACAACGTGGAAGCCAAGCGACTGAACGATGAGGCTCGTaa                                                                                                                                            <  1:461009/301‑1 (MQ=255)`  
                                                                                                                                                                         `ggTATCGCTGGCTACATGGAATACAACAAGGAAACCGGATGGCTTGAACCATCAAGTTACTCAGGGGAAGAAGAGTCACACTCAGAGTCAACAGACTGGTCCAACGACACTGACTTCTGACAGGATTCTTGAcagttgtttcatatgaagagattgttaagtcacgataatcaataggagaaatcaatatgatcgtttctgacatcgaagctaacgccctcttagagagcgtcactaagttccactgcggggttatctacgactactcccccgctgagtacgtaagctaccgtccgagtga                                                                                                                                   >  1:200228‑M1/1‑132 (MQ=255)`  
                                                                                                                                                                                  `ggCTACATGGAATACAACAAGGAAACCGGATGGCTTGAACCATCAAGTTACTCAGGGGAAGAAGAGTCACACTCAGAGTCAACAGACTGGTCCAACGACACTGACTTCTGACAGGATTCTTGATGACTTTCCAGACGACTACGAGAAGTTTCGCTGGAGAGTCCCATTCTAATACGACTCACTAAAGGAGACACACCATGTTCAAACTGATTAAGAAGTTAGGCCAACTGCTGGTTCGTATGTACAACGTGGAAGCCAAGCGACTGAACGATGAGGCTCGTaactgtctcttatacacatt                                                                                                                          >  1:295860/1‑283 (MQ=255)`  
                                                                                                                                                                                   `gCTACATGGAATACAACAAGGAAACCGGATGGCTTGAACCATCAAGTTACTCAGGGGAAGAAGAGTCACACTCAGAGTCAACAGACTGGTCCAACGACACTGACTTCTGACAGGATTCTTGAcagttgtttcatatgaagagattgttaagtcacgataatcaataggagaaatcaatatgatcgtttctgacatcgaagctaacgccctcttagagagcgtcactaagttccactgcggggttatctacgactactccaccgctgagtacgtaagctaccgtccgagtgacttcggctgt                                                                                                                         >  1:452199‑M1/1‑122 (MQ=255)`  
                                                                                                                                                                                        `aTGGAATACAACAAGGAAACCGGATGGCTTGAACCATCAAGTTACTCAGGGGAAGAAGAGTCACACTCAGAGTCAACAGACTGGTCCAACGACACTGACTTCTGACAGGATTCTTGATGACTTTCCAGACGACTACGAGAAGTTTCGCTGGAGAGTCACATTCTAATACGACTCACTAAAGGAGACACACCATGTTCAAACTGATTAAGAAGTAAGGCCCACTGCTGGTTCGTATGTACAACGTGGAAGCCAAGCGACTGAACGATGAGGCTCGAAAAGAGGCCACACAGTCACGCGctct                                                                                                                    >  1:325648/1‑301 (MQ=255)`  
                                                                                                                                                                                               `acaacaAGTAAACCGGCTGGCTTTAACCAGCAAGTTACTCATGGGAAGAAGATTCAAACTCAGAGTCAACAGACTGGTCCAACGACACGGACTTCTGACAGGAGTCTTGATTACTTTCCAGACGACTACGAGAAGTTTCGCTGGAGAGTCCCATTCTAATACGACTCACTAAATGAGACACACCCTGTTCAAACTGATTAAGAAGTTAGGCCAACTGCTGGTTCGTATGTACAACGTGGAAGCCAAGCGACTGAACGATGAGGCTCGTAAAGAGGCCACACAGTCACGCGCTCTGGCGAtt                                                                                                             <  1:110426/301‑1 (MQ=255)`  
                                                                                                                                                                                                `aaacaaTGAAACCGGATGGCTTGAACCATCAAGTTACTCAGGCGAAGAAGAGTCACTCTCAGAGTCAACAGACTGGTCCAACGACACTGACTTCTGACAGGATTCTTGATGACTTTCCATACAACTACGATCATTTTCGCTGGAGAGTCACATTCTAATACGACTCACTAAAGGAGACACACCATGTTCAAACTGATTAAGAAGTTAGGCCAACTGCTGGTTCGTATGTACAACGTGGAAGCCAAGCGACTGAACGATGATGCTCGTAAAGAGGCCACACAGTCACGCGCTCTGGCGATTc                                                                                                            <  1:337918/300‑1 (MQ=255)`  
                                                                                                                                                                                                    `aaGGAAACCGGATGGCTTGAACCATCAAGTTACTCAGGGGAAGAAGAGTCACACTCAGAGTCAACAGACTTGTCGAACGACACTGACTTCTGACAGGATTCTTGATGACTTTCCAGACGACTACGAGAAGTTTCGATGGAGAGTCACATTCTAATACGACTCAATAAAGTAGCCACACCATGTTCAAACTGATGAAGAAGTTAGGCCAACTGCTGGTTCGTATGTACAACGTGGAAGTCAAGCGACTGAACGATGAGGCTCGTAAATAGGCCACAAAGTCACGCGCTCTGGAGATTCgggc                                                                                                        >  1:248098/1‑298 (MQ=255)`  
                                                                                                                                                                                                       `gAAACCGGATGGCTTGAACCATCAAGTTACTCAGGGGAAGAAGAGTCACACTCAGAGTCAACAGACTGGTCCAACGACACTGACTTCTGACAGGATTCTTGAcagttgtttcatatgaagagattgttaagtcacgataatcaataggagaaatcaatatgatcgtttctgacatcgaagctaacgccctcttagagagcgtaactaagttccactgcgggattatctacgactactccaccgctgagtacgtaagctaccgtccgagtgacttcggtgcgtaactggatgcgctggaagc                                                                                                     >  1:311868‑M1/1‑102 (MQ=255)`  
                                                                                                                                                                                                            `cGGATGGCTTGAACCATCAAGTTACTCAGGGGAAGAAGAGTCACACTCAGAGTCAACAGACTGGTCCAACGACACTGACTTCTGACAGGATTCTTGATGAATTTCCAGACGACTACGAGAAGTTTCGCTGGAGAGTCCCATTCTAATACGACTCACTAAAGGAGACACACCATGTTCAAACTGATTAAGAAGTTAGGCCAACTGCTGGTTCGTATGTACAACGTGGAAGCCAAGCGACTGAACGATGAGGCTCGTAAAGAGGCCACACAGTCACGCGCTCTGGCGATTCGCTCCAACGAAc                                                                                                <  1:129833/301‑1 (MQ=255)`  
                                                                                                                                                                                                                `tGGTTTGAAACATCAGTGTACTCAGGGGAACAAGAGTCACACTCATAGTCAACCGACTGCGCCAACGACACTGACTTCTGACAGGAATCTTGCTGACTTTCCAGACGATTACGAGAAGTTTCGCTGGGGAGTCCCATTCTAATACGACTCACTAAAGGAGACACACCATGTTCAAACTGATTAAGAAGTTAGGCCAACTGCTGGTTCGTATGTACAACGTGGAAGCCAAGCGACTGAACGATGAGGCTCGTAAAGAGGCCACACAGTCACGCGCTCTGGCGATTCGCTCCAACGAACTGGc                                                                                            <  1:163073/301‑1 (MQ=255)`  
                                                                                                                                                                                                                  `gCTTGAACCATCAAGTTACTCAGGGGAAGAAGAGTCACACTCAGAGT‑‑‑CAGACTTGTCCAACGACACTGACTTC‑‑‑‑‑‑‑‑‑‑‑‑TGAcagttgtttcatatgaagagattgttaagtcacgataatcaataggagaaatcaatatgatcgtttctgacatcgaagctaacgccctcttagagagcgtcactaagttccactgcggggttatctacgactactccaccgctgagtacgtaagctaccgtccgagtgacttcggtgcgtatctggatgcgctggaagccgaggttgcacgaggcggtcttattg                                                                           <  1:221057‑M1/301‑226 (MQ=255)`  
                                                                                                                                                                                                                              `aaGTTACTCAGGGGAAGAAGAGTCACACTCAGAGTCAACAGACTGGTCCAACGACACTGACTTCTGACAGGATTCTTGATGACTTTCCAGACGACTACGATAAGTTTCGCTGGAGAGTCCCATTCTAATACGACTCACTAAAGGAGACACACCATGTTCAAACTGATTAAGAAGTTAGGCCAACTGCTGGTTCGTATGTACAACGTGGAAGCCAAGCGACTGAACGATGAGGCTCGTAAAGAGGCCACACAGTCACGCGCCCTGGCGATTCGCCCCAACGAACTGGCTGatgtctcttatt                                                                              >  1:166230/1‑290 (MQ=255)`  
                                                                                                                                                                                                                               `aGTTACTCAGGGGAAGAAGAGTCACACTCAGAGTCAACAGACTGGTCCAACGACACTGACTTCTGACAGGATTCTTGAcagttgtttcatatgaagagattgttaagtcacgataatcaataggagaaatcaatatgatcgtttctgacatcgaagctaacgccctcttagagagcgtcactaagttccactgcggggttatctacgtctactccaccgctgagtacgtaagctaccgtccgagtgacttcggtgcgtatctggatgctctggaagccgaggttgcacgaggcggtcttat                                                                             >  1:264257‑M1/1‑78 (MQ=255)`  
                                                                                                                                                                                                                                    `aagaGGGGAAGAATAGTCAAACTCAGAGTCAACAGACTGAGCCAACGACACTGACTTGTGACAAGATTCTTGATGACTTTCCAGACGACGACGAGAAGTTTCGCTGGAGAGTCCCATTATAATACGACTCACTAAAGGAGACACACCATGTTCCAACTGATTAAGAAGTTAGACCAACTGCTGGTTCGTATGTACAACGTGGAAGCCAAGCGACTGAGCGATGAGTCTCGTAAAGAGGCCACACAGTCACGCGCTCTGGCGATTCGCTCCAACGAACTGGCTGACAGTGCATCCACTAAAg                                                                        <  1:211212/298‑1 (MQ=255)`  
                                                                                                                                                                                                                                          `gggagatgtgtataagagacagTCAACAGACTTGTCCAACGACACTGACTTCTGACAGGATTCTTGATGACTTTCCAGACGACTACGAGAAGTTTCGCTGGAGAGTCCCATTCTAATACGACTCACTAAAGGAGACACACCATGTTCAAACTGATTAAGAAGTTAGGCCAACTGCTGGTTCGTATGTACAACGTGGAAGCCAAGCGACTGAACGATGAGGCTCGTAAAGAGGCCACACAGTCACGCGCTCTGGCGATTCGCTCCAACGAACTGGCTGACAGTGCATCCACTAAAGTTACCg                                                                  <  1:384473/281‑1 (MQ=255)`  
                                                                                                                                                                                                                                          `ggAAGAAGAGTCACACTCAGAGTCAACAGACTGGTCCAACGACACTGACTTCTGACAGGATTCTTGATGACTTTCCAGACGACTACGAGAAGTTTCGCTGGAGAGTCCCATTCTAATACGACTCACTAAAGGAGACACACCATGTTCAAACTGATTAAGAAGTTAGGCCAACTGCTGGTTCGTATGTACAACGTGGAAGCCAAGCGACTGAACGATGATGCTCGTAAAGAGGCCACACAGTCACGCGCTCTGGCGATTCGCTCCAACGAACTGGCTGACAGTGCATCCACTAAAGTTACCg                                                                  <  1:355033/301‑1 (MQ=255)`  
                                                                                                                                                                                                                                            `aagaagAGTCACACTTAGAGTCAACAGACTGGTCCAACGACACTGACTTCTGACAGGATTCTTGATGACTTTACAGACGACTAAGAGAAGTTTCGCTGGAGAGTCCCATTCTAATACGACTCACGAAAGGAGACACACCATGTTCAAACTGATTAAGAAGTTAGGCCAACTGCTGGTTCGTATGTACAACGTGGAAGCCAAGCTACTGAACGATGAGGCTCGTAAAGAGGCCACACAGTCACGCGCTCTGGCGATTCGCTCCAACGAACTGGCTGACAGTTCATCCACTAAAGTTACCGAg                                                                <  1:367831/301‑1 (MQ=255)`  
                                                                                                                                                                                                                                                 `gagTCACACTCAGAGTCAACAGACTGGTCCAACGACACTGACTTCTGACAGGATTCTTGATGACTTTCCAGACGACTACGAGAAGCTTCGCTGGATAGTCCCATTCTAATACGACCCACTAACGGAGCCACACCATGTTCAAACTTATT‑AGAAGTTCGGCCAAATGATGGTACGGCTGTACAACGTGGAAGCCAAGCGAATGAACGAAGAGGCTCGTAAAAAGGCCACACAGTCAATCGCCCTGGCGATTCGAACAAACGAACCGGCTGAAAGTGCAGACACCAAAGTAAcacaggtacat                                                          >  1:141723/1‑291 (MQ=255)`  
                                                                                                                                                                                                                                                   `gTCACAGTCAGAGTCAACAGACTGGTCCAACGACACTGACTTCTGACAGGATTCTTGATGACTTTCCAGACGACTACGAGAAGTTTCGCTGGAGAGTCCCATTCTAATACGACTCACTAAAGGAGACACACCATGTTCAAACTGATTAAGAAGTTAGGCCAACTGCTGGTTCGTATGTACAACGTGGAAGCCAAGCGACTGAACGATGAGGCTCGTAAAGAGGCCACACAGTCACGCGCTCTGGCGATTCGCTCCAACGAACTGGCTGACAGTGCATCCACTAAAGTTACCGAGGCTGccc                                                         <  1:155612/301‑1 (MQ=255)`  
                                                                                                                                                                                                                                                   `gTCACACTCAGAGTCAACAGACTGGTCCAACGACACTGACTTCTGACAGGATTCTTGAcagttgtttcatatgaagagattgttaagtcacgataatcaataggagaaatcaatatgatcgtttctgacatcgaagctaacgccctcttagagagcgtcactaagttccactgcggggttatctacgactactccaccgctgaggacgtaagcaaccgtccgagtgacttcggtgcgtatctggatgctctggaagccgaggttgcacgaggcggtcttattgtgttccacaacggtcaca                                                         >  1:285635‑M1/1‑58 (MQ=255)`  
                                                                                                                                                                                                                                                          `tCAGAGTCAACAGACTGGTCCAACGACACTGACTTCTGACAGGATTCTTGATGACTTTCCAGACGACTACGAGAAGTTTCGCTGGAGAGTCCCATTCTAATACGACTCACTAAAGGAGACACACCATGTTCAAACTGATTAAGAAGTTAGGCCAACTGCTGGTTCGTATGTACAACGTGGAAGACAAGCGACTGAACGATGAGGCTCGTAAAGAGGCCACACAGTCACGCGCTCTGGCGATTCGCTCCAACGAACTGGCTGACAGTGCATCCACTAAAGTTACCGAGGCTGCCCGtgtgtc                                                  <  1:174355/301‑3 (MQ=255)`  
                                                                                                                                                                                                                                                             `gagTCAACAGACTGGTCCAACGACACTGACTTCTGACAGGATTCTTGAcagttgtttcatatgaagagattgttaagtcacgataatcaataggagaaatcaatatgatcgtttctgacatcgaagctaacgccatcttagagagcgtcactaagttccactgcggggttatctacgactactccaccgctgagtacgtaagctaccgtccgagtgacttcggtgcgtatctggatgcgctggaagccgaggttgcacgaggcggtcttattgtgttccacaacggtcacaagtatgacgt                                               <  1:436539‑M1/301‑254 (MQ=255)`  
                                                                                                                                                                                                                                                             `gagTCAACAGACTGGTCCAACGACACTGACTTCTGACAGGATTCTTGATGACTTTCCAGACGACTACGAGAAGTTTCGCTGGAGAGTCCCATTCTAATACGACTCACTAAAGGAGACACACCATGTTCAAACTGATTAAGAAGTTAGGCCAACTGCTGGTTCGTATGTACAACGTGGAAGCCAAGCGACTGAACGATGAGGCTCGTAAAGAGGCCACACAGTCACGCGCTCTGGCGATTCGCTCCAAAGAACTGGCTGACAGTGCATCCACTAAAGTTACCGAGGCTGCCCGTGTGGCaaa                                               >  1:168660/1‑301 (MQ=255)`  
                                                                                                                                                                                                                                                               `gTCAACAGACTGGTCCAACGACACTGACTTCTGACAGGATTCTTGATGACTTTCCAGACGACTACGAGAAGTTTCGCTGGAGAGTCCCATTCTAATACGACTCACTAAAGGAGACACACCATGTTCAAACTGATTAAGAAGTTAGGCCAACTGCTGGTTCGTATGTACAACGTGGAAGCCAAGCGACTGAACGATGAGGCTCGTAAAGAGGCCACACAGTCACGCGCTCTGGCGATTCGCTCCAACGAACTGGCTGACAGTGCATCCACTACAGTTACCGAGGCTGCCCGTGTGGCAAAcc                                             >  1:307516/1‑301 (MQ=255)`  
                                                                                                                                                                                                                                                               `gTCAACAGACTGGTCCAACGACACTGACTTCTGACAGGATTCTTGATGACTTTCCAGACGACTACGAGAAGTTTCGCTGGAGAGTCCCATTCTAATACGACTCACTAAAGGAGACACACCATGTTCAAACTGATTAAGAAGTTAGGCCAACTGCTGGTTCGTATGTACAACGTGGAAGCCAAGCGACTGAACGATGAGGCTCGTAAAGAGGCCACACAGTCACGCGCTCTGGCGATTCGCTCCAACGAACTGGCTGACAGTGCATCCACTAAAGTTACCGAGGCTGCCCGTGTGGCAAAcc                                             >  1:398652/1‑301 (MQ=255)`  
                                                                                                                                                                                                                                                                          `ggTCCAACGACACTGACTTCTGACAGGATTCTTGAcagttgtttcatatgaagagattgttaagtcacgataatcaataggagaaatcaatatgatcgtttctgacatcgaagctaacgccctcttagagagcgtcactaagttccactgcggggttatctacgactactccaccgctgagtacgtaagctaccgtccgagtgacttcggtgcgtatctggatgcgctggaagccgaggttgcacgaggcggccttattgtgttccacaacggtcacaagtatgactgtctcttatcccca                                  >  1:291007‑M1/1‑35 (MQ=255)`  
                                                                                                                                                                                                                                                                          `ggTCCAACGACACTGACTTCTGACAGGATTCTTGATGACTTTCCAGACGACTACGAGAAGTTTCGCTGGAGAGTCCCATTCTAATACGACTCACTAAAGGAGACACACCATGTTCAAACTGATTAAGAAGTTAGGCCAACTGCTGGTTCGTATGTACAACGTGGAAGCCAAGTGACTGAACGATGAGGCTCGTAAAGAGGCCACACAGTCACGCGCTCTGGCGATTCGCTCCAACGAACTGGCTTACAGTGCATCCACTAAAGTTACCGAGGCTGCCCGTGTGGCAAACCACGCTCAACAg                                  >  1:150964/1‑301 (MQ=255)`  
                                                                                                                                                                                                                                                                          `ggTCCAACGACACTGACTTCTGACAGGATTCTTGATGACTTTCCAGACGACTACGAGAAGTTTCGCTGGAGAGTCCCATTCTAATACGACTCACTAAAGGAGACACACCATGTTCAAACTGATTAAGAAGTTAGGCCAACTGCTGGTTCGTATGTACAACGTGGAAGCCAAGCGACTGAACGATGAGGCTCGTAAAGAGGCCACACAGTCACGCGCTCTGGCGATTCGCTCCAACGAACTGGCTGACAGTGCATCCACTAAAGTTACCGAGGCTGCCCGTGTGGCAAACCAAGCTCAACAg                                  >  1:37909/1‑301 (MQ=255)`  
                                                                                                                                                                                                                                                                           `gTCCAACGACACTGACTTCTGACAGGATTCTTGATGACTTTCCAGACGACTACGAGAAGTTTCGCTGGAGAGTCCCATTCTAATACGACTCACTAAAGGAGACACACCATGTTCAAACTGATTAAGAAGTTAGGCCAACTGCTGGTTCGTATGTACAACGTGGAAGCCAAGCGACTGAACGATGAGGCTCGTAAAGAGGCCACACAGTCACGCGCTCTGGCGATTCGCTCCAACGAACTGGCTGACAGTGCATCCACTAAAGTTACCGAGGCTGCCCGTGTGGCAAACCAAGCTCAACAGc                                 <  1:374855/301‑1 (MQ=255)`  
                                                                                                                                                                                                                                                                                   `acacTGACTTCTGACAGGATTCTTGATGACTTTCCAGACGACTACGAGAAGTTTCGCTGGAGAGTCCCATTCTAATACGACTCACTAAAGGAGACACACCATGTTCAAACTGATTAAGAAGTTAGGCCAACTGCTGGTTCGTATGTACAACGTGGAAGCCAAGCGACTGAACGATGAGGCTCGTAAAGAGGCCACACAGTCACGCGCTCTGGCGATTCGCTCCAACGAACTGGCTGACAGTGCATCCACTAAAGTTACCGAGGCTGCCCGTGTGGCAAACCAAACTCACAAGCTTTCCaaa                         >  1:3940/1‑301 (MQ=255)`  
                                                                                                                                                                                                                                                                                      `cTGACTTCTGACAGGATTCTTGATGACTTTCCAGACGACTACGAGAAGTTTCGCTTGAGAGTCCCATTCTAATACGACTCACTAAAGGAGACACACCATGTTCAAACTGATTAAGAAGTTAGGCCAACTGCTGGTTCGTATGTACAACGTGGAAGCCAAGCGACTGAACGATGAGGCTCGTAAAGAGGCCACACAGTCACGCGCTCTGGCGATTCGCTCCAACGAACTGGGTGACAGTGCATCCACTAAAGTTACCGAGGCTGCCCGTGTGGCAAACCAAGCTCAACAGCTTTCCAAATTc                      <  1:85654/301‑1 (MQ=255)`  
                                                                                                                                                                                                                                                                                        `gACTTCTGACAGGATTCTTGATGACTTTCCAGACGACTACGAGAAGTTTCGCTGGAGAGTCCCATTCTAATACGACTCACTACAGGAGACACACCATGTTCAAACTGATTAAGAAGTTAGGCCAACTGCTGGTTCGTATGTACAACGTGGAAGCCAAGCGACTGAACGATGAGGCTCGTAAAGAGGCCACACAGTCACGCGCTCTGGCGATTCGCTCCAACGAACTGCCTGACAGTGCATCCACTAAAGTTACCGAGGCTGCCCGTGTGGCAAACCAAGCTCAACAGCTTTCCACATTCtt                    >  1:317029/1‑301 (MQ=255)`  
                                                                                                                                                                                                                                                                                          `cTTCTGACAGGATTCTTGATGACTTTCCAGACGACTACGAGAAGTTTCGCTGGAGAGTCCCATTCTAATACGACTCACTAAAGGAGACACACCATGTTCAAACTGATTAAGAAGTTAGGCCAACTGCTGGTTCGTATGTACAACGTGGAAGCCAAGCGACTGAACGATGATGCTCGTAAAGAGGCCACACAGTCACGCGCTCTGGCGATTCGCTCCAACGAACTGGCTGACAGTGCATCCACTAAAGTTACCGAGGCTGCCCGTGTGGCAAACCAAGCCCAACAGCTTTCCAAATTCtttt                  >  1:404919/1‑300 (MQ=255)`  
                                                                                                                                                                                                                                                                                             `cTGACAGGATTCTTGAcagttgtttcatatgaagagattgttaagtcacgataatcaataggagaaatcaatatgatcgtttctgacatcgaagctaacgccctcttagagagcgtcactaagttccactgcggggttatctacgactactccaccgctgagtacgtaagctaccgtccgagtgacttcggtgcgtatctggatgcgctggaagccgaggttgcacgaggcggtcttattgtgttccacaacggtcacaagtatgacgttcctgcattgaccaaactggcaaagttgcaat               <  1:427810‑M1/301‑286 (MQ=255)`  
                                                                                                                                                                                                                                                                                                        `cTTGATTACTTTCCAGACGACTACGAGAAGTTTCGCTGGAGAGTCCCATTCTAATACGACTCACTAAAGGAGACACACCATGTTCAAACTGATTAAGAAGTTAGGCCAACTGCTGGTTCGTATGTACAACGTGGAAGCCAATCGACTGAACGATGAGGCTCGTAAAGAGGCCACACAGTCACGCGCTCTGGCGATTCGCTCCAACGAACTGGCTGACAGTGCCTCCACTAAAGTTACCGAGGCTGCCCGTGTGGCAAACCAAGCTCAACAGCTTTCCAAATTCTTTTAGTAATCAAACAgg    >  1:114103/1‑301 (MQ=255)`  
                                                                                                                                                                                                                                                                                                          `tgatgaCTTTCCAGACGACTACGAGAAGTTTCGCTGGAGAGTCCCATTCTAATACGACTCACTAAAGGAGACACACCATGTTCAAACTGATTAAGAAGTTAGGCCAACTGCTGGTTCGTATGTACAACGTGGAAGCCAAGCGACTGAACGATGAGGCTCGTAAAGAGGCCACACAGTCACGCGCTCTGGCGATTCGCTCCAACGAACTGGCTGACAGTGCATCCACTAAAGTTACCGAGGCTGCCAGTGTGGCAAACCAAGCTCAACACCTTTCCAAATTCTTTGAGTAATCAAACAGgag  <  1:217518/301‑1 (MQ=255)`  

                                                                                                                                                                                                                                                                                                            `|`                                                                                                                                                                                                                                                                                                            
`AGGTAAAGCACATGAGGAAGGTCGCCCCGTTTCTATTACTGACCTACGTGGTTCTGGCGCACTACGCCAACTATCTGATACTATTATTGCCCTTGAGCGTAATCAGCAAGGCGATATGCCTAACCTTGTCCTCGTTCGTATTCTCAAGTGCCGCTTTACTGGTGATACTGGTATCGCTGGCTACATGGAATACAACAAGGAAACCGGATGGCTTGAACCATCAAGTTACTCAGGGGAAGAAGAGTCACACTCAGAGTCAACAGACTGGTCCAACGACACTGACTTCTGACAGGATTCTTGATGACTTTCCAGACGACTACGAGAAGTTTCGCTGGAGAGTCCCATTCTAATACGACTCACTAAAGGAGACACACCATGTTCAAACTGATTAAGAAGTTAGGCCAACTGCTGGTTCGTATGTACAACGTGGAAGCCAAGCGACTGAACGATGAGGCTCGTAAAGAGGCCACACAGTCACGCGCTCTGGCGATTCGCTCCAACGAACTGGCTGACAGTGCATCCACTAAAGTTACCGAGGCTGCCCGTGTGGCAAACCAAGCTCAACAGCTTTCCAAATTCTTTGAGTAATCAAACAGGAG  >  NC_001604.1/12977‑13575`

  

Alignment Legend

Aligned base mismatch/match (shaded by quality score): `ATCG/ATCG` `< 3 ≤` `ATCG/ATCG` `< 10 ≤` `ATCG/ATCG` `< 19 ≤` `ATCG/ATCG` `< 34 ≤` `ATCG/ATCG` `< 38 ≤` `ATCG/ATCG`

Unaligned base: `atcg`    Masked matching base: `atcg`    Alignment gap: `‑`    Deleted base: `‑`

Reads not counted as support for junction

read\_name Not counted due to insufficient overlap past the breakpoint.

read\_name Not counted due to not crossing MOB target site duplication.
